# Supplementary material for: Long-Term Outcomes of Tachycardia-Induced Cardiomyopathy Compared with Idiopathic Dilated Cardiomyopathy
Source: J Clin Med. 2023 Feb 10;12(4):1412. doi: 10.3390/jcm12041412 (PMC9960677; doi:10.3390/jcm12041412)
Supplement: Supplementary file 1 [file jcm-12-01412-s001.zip › jcm-2156661-supplementary.pdf]

Long-term outcomes of tachycardia induced cardiomyopathy compared with idiopathic dilated cardiomyopathy

Moshe Katz*[1,2,3], Amit Meitus[2], Michael Arad[1,2], Anthony Aizer[3], Eyal Nof[1,2] and Roy Beinart[1,2]

[1]Sheba Medical Center, Ramat Gan, Israel
[2]Sackler School of Medicine, Tel-Aviv University, Israel
[3]NYU Grossman School of Medicine, New York, USA

Table S1. History of arrhythmia and rhythm at presentation.

	IDCM N=66	TICMP N=64
History of atrial fibrillation; n (%)	1 (1.5)	17 (27)
Rhythm at presentation		
Sinus w/o arrhythmia; n (%)	61 (90)	0
Atrial fibrillation; n (%)	3 (5)	49 (76)
Atrial flutter; n (%)	2 (3)	8 (13)
Atrial tachycardia; n (%)	0	1 (2)
SVT; n (%)	0	2 (3)
VPBs; n (%)	0	4 (6)
Catheter-ablation; n (%)	0	12 (19)

IDCM- idiopathic dilated cardiomyopathy; SVT- supraventricular tachycardia; TICMP- tachycardia induced cardiomyopathy; VPBs- ventricular premature beats; w/o without.

Table S2. Devices implanted and echocardiographic parameters collected during follow-up.

	IDCM N=66	TICMP N=64	p-value
PM implantation; n (%)	1 (1.5)	12 (19)	0.001*
CRTP	0	3 (5)	
Single chamber	0	4 (6)	
Dual chamber	1 (1.5)	5 (8)	
AICD implantation; n (%)	23 (35)	3 (5)	<0.001*
CRTD	14 (21)	1 (2)	
Single chamber	8 (12)	0	
Dual chamber	1 (2)	2 (3)	
LVEF, %; median [IQR]	35 [20-45]	55 [47-60]	<0.001*
LVEDD, cm; median [IQR]	5.5 [5-6.1]	5.2 [4.7-5.6]	0.009*
LVEDS, cm; median [IQR]	4.3 [3.8-5.5]	3.6 [3.3-4]	<0.001*
LA diameter, cm; median [IQR]	4.1 [3.6-4.6]	4.4 [4-4.7]	0.015*
LV mass, g; median [IQR]	209 [181-266]	193 [159-246]	0.095
SPAP, mmHg; median [IQR]	29 [27-37]	33 [28-43]	0.068

AICD- automated implantable cardioverter defibrillator; CRTD- Cardiac Resynchronization Therapy Defibrillator; CRTP- Cardiac Resynchronization Therapy Pacemaker; IDCM- idiopathic dilated cardiomyopathy; IQR- interquartile range; LA-left atrium; LVEDD-left ventricular end-diastolic diameter; LVEF-left ventricular ejection fraction; LVESD-left ventricular end-systolic diameter; PM- pacemaker; SPAP-systolic pulmonary artery pressure ;TICMP- tachycardia induced cardiomyopathy; Chi-square or Fisher's exact tests were performed for categorical parameters. Mann–Whitney test was performed for continuous parameters. Significant p-value was marked by *.
